# Low Genetic Quality Alters Key Dimensions of the Mutational Spectrum

**DOI:** 10.1371/journal.pbio.1002419

**Published:** 2016-03-25

**Authors:** Nathaniel P. Sharp, Aneil F. Agrawal

**Affiliations:** 1 Department of Ecology and Evolutionary Biology, University of Toronto, Toronto, Ontario, Canada; 2 Department of Zoology, University of British Columbia, Vancouver, British Columbia, Canada; Institute of Science and Technology Austria (IST Austria), AUSTRIA

## Abstract

Mutations affect individual health, population persistence, adaptation, diversification, and genome evolution. There is evidence that the mutation rate varies among genotypes, but the causes of this variation are poorly understood. Here, we link differences in genetic quality with variation in spontaneous mutation in a *Drosophila* mutation accumulation experiment. We find that chromosomes maintained in low-quality genetic backgrounds experience a higher rate of indel mutation and a lower rate of gene conversion in a manner consistent with condition-based differences in the mechanisms used to repair DNA double strand breaks. These aspects of the mutational spectrum were also associated with body mass, suggesting that the effect of genetic quality on DNA repair was mediated by overall condition, and providing a mechanistic explanation for the differences in mutational fitness decline among these genotypes. The rate and spectrum of substitutions was unaffected by genetic quality, but we find variation in the probability of substitutions and indels with respect to several aspects of local sequence context, particularly GC content, with implications for models of molecular evolution and genome scans for signs of selection. Our finding that the chances of mutation depend on genetic context and overall condition has important implications for how sequences evolve, the risk of extinction, and human health.

## Abstract 2

The genome sequences of *Drosophila* mutation accumulation lines suggest that low-quality genotypes repair genetic damage using more error-prone mechanisms; mutation rates vary with both individual condition and local sequence context.

## Author Summary

The replication and maintenance of genomes is essential to all organisms, and multiple cellular pathways serve to correct replication errors and repair DNA damage. The use of these repair pathways can vary among individuals, and we hypothesized that those in poor condition may be less capable of effectively repairing their DNA. We used genome sequencing to study new mutations in experimental fruit fly lineages, some of which had reduced condition due to previously-existing mutations in their genomes. Based on the new mutations we observed, we conclude that flies in poor condition repaired breaks in their DNA less effectively, leading to mutations that reduced the fitness of their offspring. We also found that some areas of the genome were more likely to mutate than others, altering predictions for how genome sequences evolve. If the presence of deleterious genetic variants increases the mutation rate, as our results indicate, this is expected to increase the risk of extinction in small populations, but could also accelerate adaptation to new environments. Our results further imply that individuals in poor condition are at increased risk of acquiring cancer and transmitting genetic disorders to offspring.

## Introduction

In the genomes of all organisms, there is an inescapable risk of spontaneous mutations, which are seldom beneficial. This risk is reduced by cellular mechanisms that correct replication errors and repair DNA damage. If the degree of damage or the efficacy of repair is influenced by local sequence context, genetic background, or environmental conditions, then the number and kinds of mutations that ultimately occur would be similarly influenced. There is growing recognition of the potential for extensive context-dependent mutation, including in complex eukaryotes [1].

We were particularly interested in the relationship between the spontaneous germline mutation rate and the number of deleterious alleles already present in the genome, i.e., genetic quality. If deleterious mutations are more likely to occur in genotypes that are “loaded” with deleterious alleles, then the resulting positive feedback loop would alter the equilibrium mutation rate, mean fitness, and the risk of extinction by mutational meltdown [2,3]. There is also increasing interest in the effects of local sequence context on mutation within genomes, which will allow for more realistic models of neutral molecular evolution. Such models are interesting in their own right and as a basis for null models in genome scans for signs of selection [4,5].

To investigate the effect of genetic quality on spontaneous germline mutation, we conducted a mutation accumulation (MA) experiment using *Drosophila melanogaster* in which focal 2nd chromosomes accumulated spontaneous mutations over 52 generations in the presence of either a wild-type “unloaded” 3rd chromosome or a 3rd chromosome “loaded” with known deleterious alleles. At regular intervals, we extracted focal chromosomes from the different backgrounds and assessed their fitness in a common genetic background. As previously reported, we found that focal chromosomes maintained in the loaded backgrounds declined in fitness almost three times faster than those maintained in unloaded backgrounds, providing indirect evidence that deleterious mutations arose at a greater rate in low-quality genotypes [6]. In addition, the effect of our genetic quality manipulations on mutational decline was highly correlated with the effect of the manipulations on body mass, suggesting that overall condition mediated the effect of genetic quality on the mutation rate [6].

However, fitness measures do not directly reveal the number or type of mutations occurring, which may provide insight into the mechanism through which genetic quality or condition affects mutation. One possibility is that condition affects the likelihood of error during DNA replication. DNA polymerases vary in fidelity [1,7], and low condition might lead to greater use of low-fidelity polymerases, affecting the rate of single-nucleotide substitutions. Alternatively, the genome might be subject to a higher level of damage in low-condition individuals. For example, low-condition individuals could have elevated levels of endogenous mutagens, such as oxygen-centered free radicals [1,8]. However, in *Caenorhabditis elegans*, the mutagenic effect of oxidative stress seems to be minor compared to other sources of mutation [9].

Another idea is that high- and low-condition individuals could experience the same level of DNA damage but differ in the repair pathways they employ. DNA double-strand breaks (DSBs) are common and highly toxic to cells, and can be repaired through several mechanisms, which differ in their genomic consequences [10]. A DSB can be repaired by ligating the broken DNA ends through nonhomologous end-joining (NHEJ), resulting in an insertion or deletion (indel) relative to the original sequence. An alternative is homology-directed repair, which restores the original sequence surrounding a DSB by copying from a homologous template. In *D*. *melanogaster*, the homologous chromosome is often used as a template, rather than the sister chromatid, which can result in allelic gene conversion [11]. This conservative approach to repair is more time consuming [12], suggesting a possible trade-off between repair fidelity and energetic cost, and there is evidence that DNA repair is sensitive to diet quality in *Drosophila* [13,14]. We might therefore expect genetic quality to affect the prevalence of gene conversion events, which result from high-fidelity DSB repair, relative to indel mutations, which result from low-fidelity DSB repair.

Finally, in *Drosophila* and many other organisms, the movement of transposable elements (TEs) can lead to new insertions and DSBs at excision sites, with the potential for significant fitness effects [15–17]. The movement of some elements can be regulated by the host genome, and there is evidence that stress, particularly in the form of high temperature, increases TE mobilization [18]. This suggests the possibility of different rates of TE insertions in individuals of different conditions.

To explore these possibilities, we sequenced focal chromosomes from 112 of our MA lines, which accumulated mutations in seven different backgrounds (one unloaded, six loaded) for 52 generations, for a total of 5,824 MA generations. We used stringent criteria to call mutations in 38 pooled samples and estimated our power to detect true mutations given these criteria (S1 Data). We compared the rates of several types of mutations between our loaded and unloaded treatments. In addition to mutational differences between genomes, we also examined the relationship between mutation rate and sequence context at multiple spatial scales.

## Results

**Fig 1:**
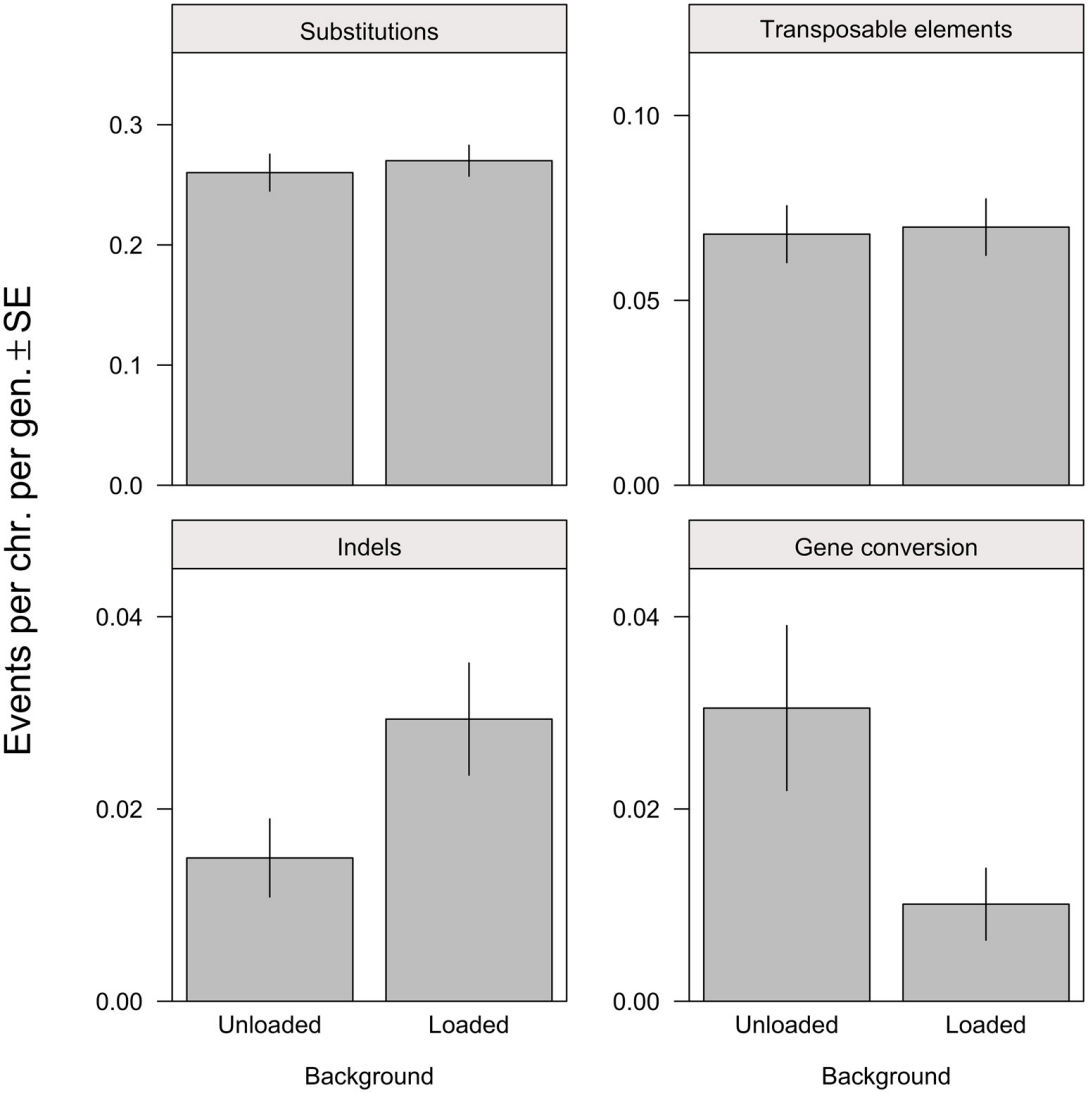
Rates of mutation and gene conversion on unloaded and loaded backgrounds (predicted values per 2nd chromosome per generation). There was no difference between treatments in the rate of substitution (Generalized Linear Model [GLM]: *Z* = 0.51, *p* = 0.612) or TE insertion (GLM: *Z* = 0.20, *p* = 0.840). The rate of indel mutation was significantly higher in loaded lines (GLM: *Z* = 2.17, *p* = 0.030), and the rate of gene conversion was significantly higher in unloaded lines (quasi-Poisson GLM: *t* = −2.41, *p* = 0.021; GLMM [generalized linear mixed model]: χ^2^ = 4.24, *p* = 0.039). See S1 Data for plot data.

**Fig 2:**
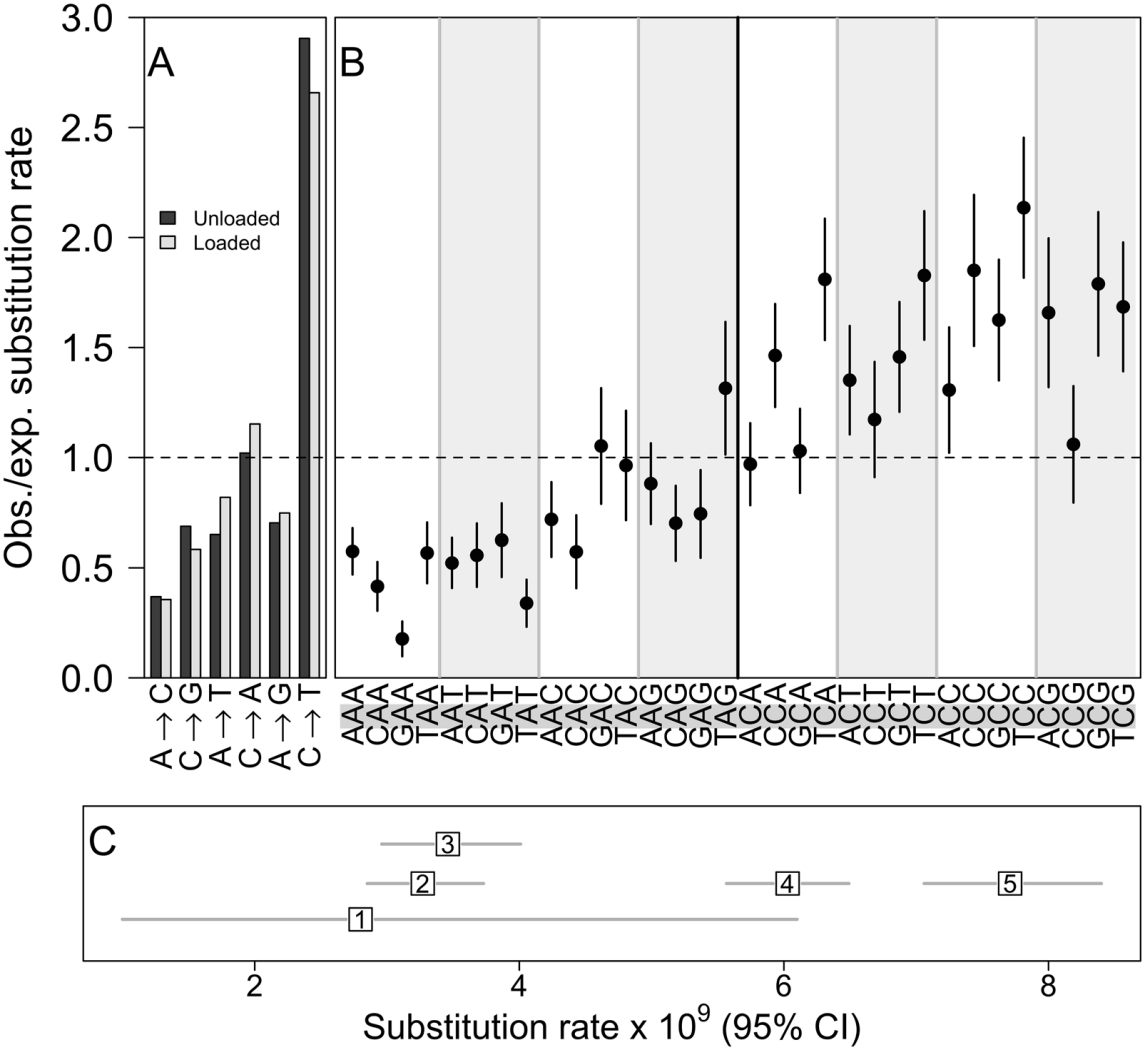
Spectrum and context dependence of single-nucleotide substitutions. (A, B) Observed substitution rates relative to the rate expected if all substitutions were equally likely. (A) The six possible substitutions occurred at unequal rates (χ^2^ = 434, *df* = 5, *p* < 1 × 10^−15^), with no difference between loaded and unloaded backgrounds (χ^2^ = 2.87, *df* = 11, *p* = 0.99). (B) Rates of substitution at the central nucleotide (highlighted in grey) for each 3-bp context, with standard error bars. Substitution rate was higher for G:C sites (paired *t* = 9.69, *df* = 15, *p* < 1 × 10^−7^) and varied significantly with 3-bp context at both A:T sites (χ^2^ = 39.24, *df* = 15, *p* < 0.001) and G:C sites (χ^2^ = 25.24, *df* = 15, *p* = 0.047), but neither differed between loaded and unloaded backgrounds (χ^2^ = 10.56, *df* = 63, *p* = 1; values shown are pooled across backgrounds). (C) Comparison of substitution rate estimates among recent studies. Our estimate (“4”) is intermediate compared with the two backgrounds studied by [19] (“2” and “5”); “1” = [20], “3” = [21]. See S1 Data for plot data.

The rate of single-nucleotide substitution in our lines was 6.03 × 10^−9^ per base pair per generation (95% confidence interval [CI]: 5.57–6.50 × 10^−9^), but there was no indication of a difference between loaded and unloaded lines (Fig 1). There was also no difference in the substitution spectrum between treatments (Fig 2A). We found that 4% of the 786 substitutions were likely the result of multinucleotide mutation events, i.e., multiple closely spaced substitutions, similar to previous observations [19].

Though substitutions are common, they may be less likely to cause fitness effects than other types of mutations. We also detected small insertions and deletions (indels) in our lines (37 deletions, 12 insertions; mean length 1.9 bp). As previously observed in flies [19,22], there is a significant deletion bias among such events (χ^2^ = 12.75, *p* < 0.001). In contrast to substitutions, indel rates were not the same across backgrounds: the rate of indel mutation was almost twice as high in loaded backgrounds compared with unloaded backgrounds (Fig 1; unloaded rate: 3.38 × 10^−10^, 95% CI 1.52–5.24 × 10^−10^; loaded rate: 6.65 × 10^−10^, 95% CI 3.98–9.32 × 10^−10^). This higher frequency of indels may have led to faster fitness decline in loaded lines, particularly if indels are more likely than substitutions to have relatively large fitness effects [23]. In accordance with this hypothesis, among mutations in coding regions, indels were significantly more likely than substitutions to be nonsynonymous (substitutions: 135/184, indels: 15/15, Fisher’s exact test: *p* = 0.024) and to generate premature stop codons (substitutions: 8/184, indels: 13/15, Fisher’s exact test: *p* = 2.38 × 10^−13^).

As intended, the accumulated mutations appear to be unbiased by selection. The observed frequency of substitutions and indels in genes (73.8%) did not differ from the neutral expectation (74.2%; binomial test: *p* = 0.84; this was also the case considering substitutions and indels separately). Indels and substitutions did not differ from one another in their likelihood of occurring in genes (indels: 80.0%, substitutions: 73.5%, Fisher’s exact test: *p* = 0.40) or in protein coding sequence (indels: 30.6%, substitutions: 24.3%, Fisher’s exact test: *p* = 0.31). Substitutions in protein coding sequence were not less likely to be nonsynonymous than expected by chance (observed: 73.4%, expected: 74.4%; χ^2^ = 0.11, *df* = 1, *p* = 0.74).

Unlike most previous MA studies of *Drosophila* where mutations accumulated in homozygotes, in our experiment the focal chromosome was maintained in a heterozygous state by crossing MA males to females from a marked stock, “*vg*.” This allowed us to detect 38 spontaneous mitotic homologous gene conversion events that occurred in these MA males (which lack meiotic recombination) by identifying regions of the focal chromosome containing substitutions matching SNPs found in the *vg* population. Previous evidence indicates that mitotic gene conversion tracts are exponentially distributed in length, with a mean of 1,463 bp [24], and the minimum tract lengths we observed (see Methods; mean 1,305 bp) did not differ from this distribution (Kolmogorov-Smirnov test, *D* = 0.134, *p* = 0.5). There was no difference in the distribution of tract lengths of events from loaded versus unloaded backgrounds (Wilcoxon rank-sum test: *W* = 123, *p* = 0.33; Kolmogorov-Smirnov test, *D* = 0.36, *p* = 0.18). The rate of gene conversion was over three times greater in the unloaded background compared with the loaded backgrounds (Fig 1).

Although male *D*. *melanogaster* lack meiotic recombination, studies using transgenic constructs indicate that homology-directed repair can sometimes result in mitotic crossing over in addition to gene conversion [11], and we found several cases of crossing over in our lines (S2 Fig). Genomic regions found to have undergone exchange with the *vg* stock were excluded from all analyses, including those described above, except the analysis focusing specifically on mitotic crossing over. Just as unloaded lines had a higher rate of gene conversion, they were also more likely to experience crossing over (unloaded: 4/52 lines tested, loaded: 1/132 lines tested, Fisher’s exact test: *p* = 0.023). We hypothesize that these crossing over events occurred during mitotic DSB repair; our data on gene conversion suggest that unloaded lines repaired DNA breaks using the homologous chromosome at a greater rate than loaded lines, which may have led to a greater rate of crossing over in the unloaded lines. According to a previous estimate, 7% of gene conversion repair events are associated with crossing over [11]. Given our estimated rates of gene conversion, and accounting for the fraction of the second chromosome where the products of crossing over would not disrupt the markers for chromosome tracking (i.e., undetected crossing over during the MA experiment), the frequencies of crossover events we observed are not significantly different from those expected given the prediction in [11] (binomial tests; unloaded: expected freq. = 8.0%, *p* = 1; loaded: expected freq. = 2.7%, *p* = 0.27). Thus, the higher rate of crossing over in the unloaded treatment is consistent with the higher rate of gene conversion in that treatment. A reanalysis of fitness measures from [6] indicates that the patterns we reported previously remain significant (and in fact become stronger) when lines with crossing over are excluded (S4 Text).

**Fig 3:**
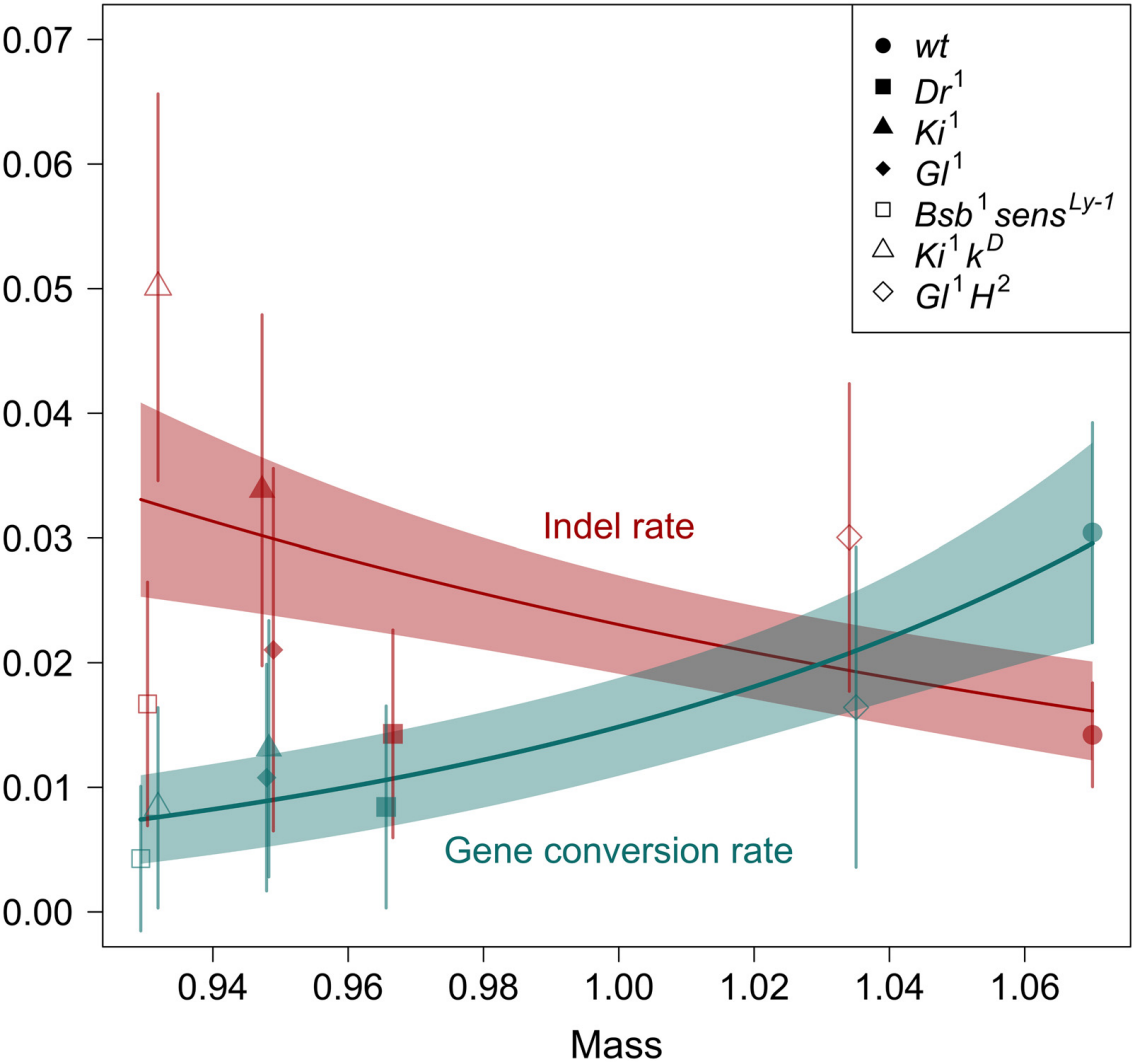
Indel and gene conversion rates are related to body mass. Rates per haploid 2nd chromosome per generation versus dry mass of males relative to a standard genotype reared in the same vial [6]. Indel rate declined significantly with body mass (GLM: *Z* = −2.12, *p* = 0.034) and gene conversion rate increased significantly with body mass (quasi-Poisson GLM: *t* = 2.42, *p* = 0.021; GLMM: χ^2^ = 4.80, *p* = 0.029). Lines and shaded regions are predicted values and standard errors from (quasi) Poisson GLMs on 38 samples. Points are predicted values for each of the seven genetic backgrounds, with horizontal jitter added for clarity. Legend indicates the treatment alleles on each genetic background (*wt* = wild-type, i.e., the unloaded treatment). See S1 Data for plot data.

In our original MA study [6], there were ten different “loaded” backgrounds, and some had larger effects on the carrier’s condition (assessed as body mass) than others. We previously reported a relationship between fitness decline in a given background and the condition of individuals with that background. A background that caused a 10% decrease in body mass was inferred to cause 2-fold faster fitness decline in the focal chromosome [6], suggesting that the effect of genetic quality on mutation rate was mediated by individual condition. Using the present dataset, which includes data from six loaded backgrounds and the unloaded background, we examined the relationship between indel or gene conversion rate and body mass across genetic backgrounds. We find that mass is a significant predictor of both types of event, with indels negatively associated with mass and gene conversion positively associated with mass (Fig 3). We similarly detect a significant relationship between mass and the difference between indel and gene conversion count per sample (*t* = –3.00, *p* = 0.005).

Certain stressors may lead to elevated transposition of mobile genetic elements [25]. We attempted to determine the number of TE insertions in our MA lines using *PoPoolationTE* [26]. We detected 226 putative insertions (S1 Data), but there was no evidence that the rate of transposition differed between treatments (Fig 1). Our ability to correct for false negatives for this type of mutation is limited (see Methods), but our best estimate of the rate of new insertions (0.069 insertions per haploid 2nd chromosome per generation; 95% CI = 0.057–0.081) is similar to previous findings obtained with very different methods when extrapolated to the haploid genome (0.17 versus 0.1–0.2; [16]).

In addition to differences between genetic quality treatments, our data also provide insight into other dimensions of variation in the mutational spectrum. The substitution rate we observed is higher than some other estimates but intermediate between the two genotypes described by [19] (Fig 2C). Those two genotypes differed from each other in their G:C to A:T transition rate and, notably, our G:C to A:T transition rate is intermediate between them (S1 Fig).

**Fig 4:**
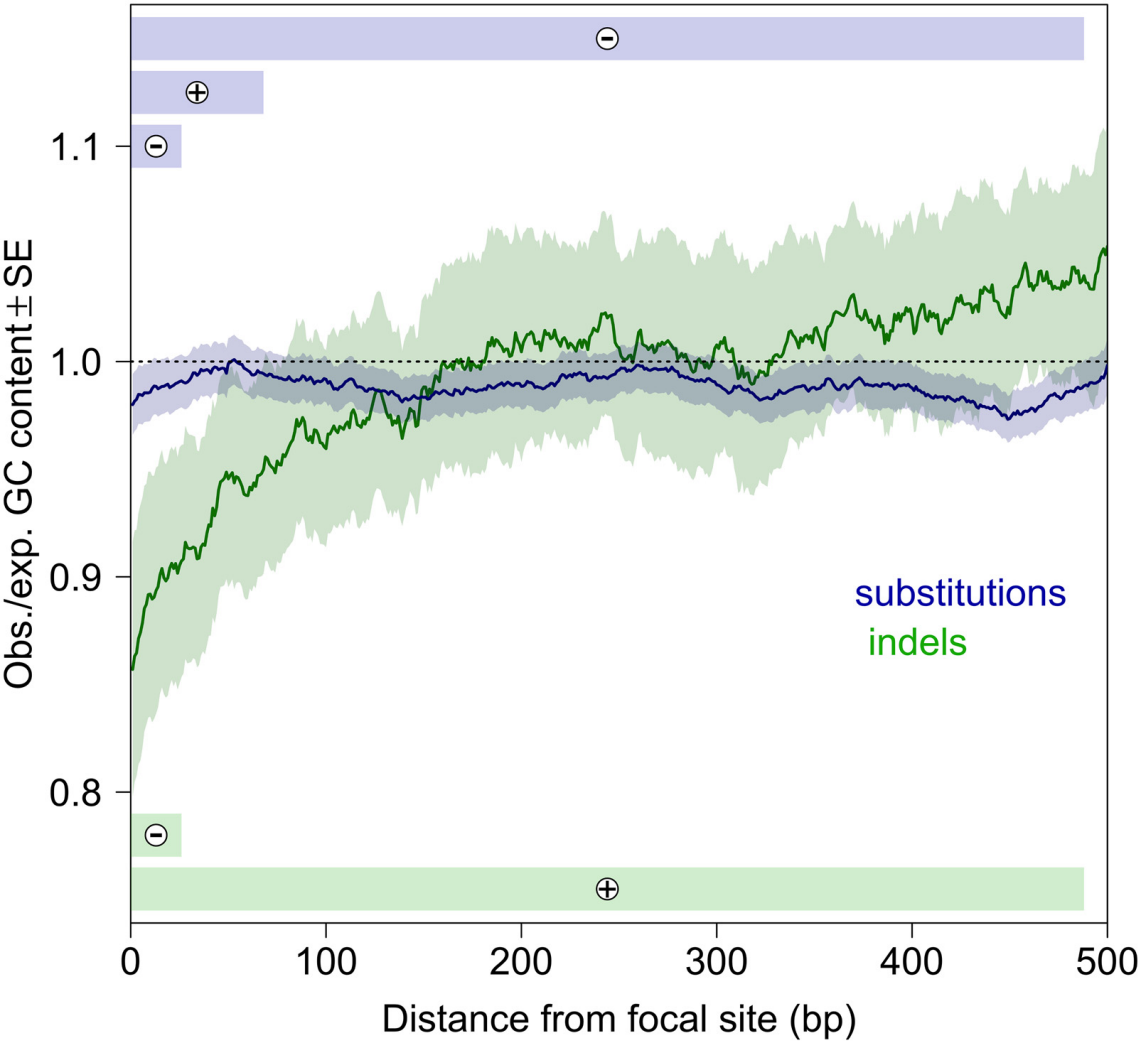
Substitution and indel rates are sensitive to local GC content. Lines represent sliding window averages of GC content surrounding mutant relative to nonmutant sites (up to 500 bp on either side), with bootstrap standard errors. Bars depict the local regions where GC content best predicts the occurrence of mutations, based on the AIC (Akaike information criterion) values of logistic models, with the sign of the effect shown on each bar. The sliding window plot does not account for the spatial correlation in GC content, whereas the logistic model does, which can explain the apparent differences. See S1 Data for plot data.

We find that the substitution rate is higher at G:C sites and influenced by adjacent bases (Fig 2B), and that substitution and indel rates are influenced by local guanine-cytosine (GC) content in different ways in exploratory statistical models (Fig 4). The best models included the GC content within three sizes of windows surrounding a focal site for substitutions but two window sizes for indels. The GC content of the window encompassing the 26 bp to the left and right of focal sites was negatively associated with the occurrence of both substitutions and indels (substitutions: *Z* = –2.88, *p* = 0.004; indels *Z* = –6.40, *p* < 1 × 10^−4^), whereas a wider region was negatively associated with substitutions (±488 bp: *Z* = –2.54, *p* = 0.011) but positively associated with indels (±498 bp: *Z* = 5.05, *p* = 0.086). Substitutions were positively associated with GC content within ±68 bp (*Z* = 2.70, *p* = 0.007). The GC content of gene conversion tracts did not differ from the random expectation (simulated: mean = 0.432, 95% CI = 0.341 − 0.543; observed: mean = 0.427, 95% CI = 0.342 − 0.480; *t* = 0.65, *p* = 0.52). A recent population genomic analysis found little evidence for GC-biased gene conversion in *D*. *melanogaster* [27]. Although our power to detect such a bias is limited, the spectrum of changes in gene conversion tracts did not differ from the neutral expectation (χ^2^ = 2.80, df = 5, *p* = 0.73; S1 Data), consistent with a lack of GC-biased gene conversion.

Dividing the chromosome into ~1-Mb windows, we found that gene conversion was more likely further from the centromere in both treatments (all data: *r*_*s*_ = 0.45, *p* = 0.002; unloaded: *r*_*s*_ = 0.33, *p* = 0.031; loaded: *r*_*s*_ = 0.34, *p* = 0.025). Combined with the positive, though nonsignificant, relationship between distance from the centromere and indel rate (*r*_*s*_ = 0.14, *p* = 0.373), this suggests that mitotic DSBs may be more likely to occur further from the centromere. An alternative explanation is that DSBs occur at equal rates across the chromosome, but those that occur closer to the centromere are less likely to get repaired, resulting in cell death, and so will go unobserved. No relationship with distance from the centromere was apparent for single-nucleotide substitutions (*r*_*s*_ = 0.02, *p* = 0.922).

## Discussion

A unique aspect of this MA study is the comparison of mutational properties of loaded and unloaded lines. Though our data is among the largest sets of de novo mutations for a nonhuman animal, mutations are rare in an absolute sense, and this limits the statistical power for making contrasts. It is worth considering the totality of the evidence for mutational differences. In comparing sequence data from loaded and unloaded lines, we have considered four types of events: single-nucleotide substitutions, indels, gene conversions, and mobile element transposition, two of which show significant rate differences (Fig 1). If the null hypothesis was true for all four event types, the chance of observing two or more significant tests (at *α* = 0.05 per test) would be *p* = 0.014, indicating we were unlikely to obtain our results by chance. Moreover, the separate analysis of mitotic crossing over using a larger set of lines provides additional support for homologous repair occurring at a higher rate in unloaded lines. Finally, the significant relationships of both indel and gene conversion rates with mass (Fig 3) add a third line of support. This third line of support is not independent of the primary tests (Fig 1) because loaded and unloaded backgrounds differ in body mass. Nonetheless, these relationships with mass could easily be nonsignificant if the primary contrasts between loaded and unloaded were significant by chance. If we limit ourselves only to the six loaded backgrounds, the point estimates for the correlations are in the expected directions (*ρ*_indel,mass_ = –0.18; *ρ*_gene_conv,mass_ = 0.51), though, unsurprisingly, nonsignificant given the small number of points. Taken together, these observations indicate important differences in mutational characteristics between flies with good and poor quality genetic backgrounds.

Indel mutations and gene conversion events are outcomes of different DSB repair pathways: repair by NHEJ results in an indel, whereas homology-directed repair results in a gene conversion event [10,12]. The contrasting pattern we observed between loaded and unloaded lines (Fig 1) suggests that these repair pathways were utilized at different rates in our experimental treatments, with the error-prone end-joining pathway employed more often in the loaded backgrounds and the conservative but time-consuming [12] homology-directed pathway employed more often in the unloaded background. A separate maximum likelihood analysis (S5 Text) indicates that the total number of indels plus gene conversion events did not differ significantly between loaded and unloaded backgrounds, suggesting that genetic quality did not affect the rate of DSBs, but rather the way in which DSBs were repaired, with gene conversion about two times more likely in the unloaded background than the loaded background.

The genome sequences of our MA lines reinforce our earlier evidence of elevated mutation rates in low-quality genotypes [6]. The rate of indels relative to gene conversion in these lines appears to be related to genetic quality (Fig 1) and overall condition (Fig 3). Condition-dependent use of alternative DSB repair pathways that differ in fidelity is a plausible explanation for these results, and for the faster fitness decline in low-quality genotypes we observed [6], but more experiments will be required to confirm this.

A previous study [14] used a reporter construct to examine how often three different pathways were used to repair induced DSBs in *D*. *melanogaster* of high versus low condition, created through a diet manipulation. In this construct, DSBs were always flanked by repeat sequences, allowing for repair via single-strand annealing (SSA), resulting in the loss of one repeat. The majority of all DSBs were repaired using SSA for both high- and low-condition flies, though more so for high-condition flies (see also [28]). For the remaining breaks, the usage rate of homologous repair (HR-h) compared to NHEJ was higher in low-condition flies than high-condition flies, although only later in life. This result seems inconsistent with our present results, but several factors complicate the comparison between studies. First, the major repair pathway in the reporter construct system, SSA, is unavailable for most spontaneous DSBs, because most do not occur between flanking repeats. Repair of DSBs is thought to be a competitive process among different pathways [29]. The availability of SSA in the construct study may substantially alter the competition between HR-h and NHEJ, and how this competition differs between high- and low-condition flies. Second, induced versus naturally-occurring DSBs could differ in their timing during the cell cycle, influencing which repair pathways are most likely to be used. Finally, the genomic location in which the reporter construct is inserted as well as the sequence of the construct itself may have some influence on repair pathway usage. While constructs are an invaluable tool, they only serve as a proxy for the subject of real interest: naturally occurring spontaneous events that lead to mutation, which are the subject of our study.

If key aspects of the mutational process are indeed sensitive to condition, this will have important consequences for populations and individuals. We have studied germline mutations, but the same DNA repair processes are used in somatic cells, suggesting that the risk of cancer-causing mutations could depend on condition [30]. These results have important implications for public health if condition—either environmentally or genetically determined—mediates the mutation rate and spectrum in humans. There is evidence for variation in germline mutation rates among human families, but the sources of this variation are uncertain [31,32]. In any organism, adaptation to new environments could be accelerated if poorly-adapted individuals are more likely to transmit new mutations to their offspring [33]. Condition-dependent mutation also has implications for the genetic benefits of mate choice and sex-biased mutation rates [34].

We identified several additional dimensions of variation in the mutational spectrum. Although the rate of substitution was not affected by our genetic quality treatment, our data support the suggestion that the rate of G:C to A:T transitions may be elevated in some *Drosophila* genotypes relative to others, increasing the overall substitution rate ([19]; Fig 2C, S1 Fig). Natural variation in mutation rates among genotypes has also been observed in nematodes [35] and algae [36]. We find that nucleotide context affects the rate of substitution at several scales, in accordance with observations from other taxa [36–39]. Beyond the elevated mutation rate at G:C sites relative to A:T sites, it is not clear to what extent these patterns are conserved. However, our finding that spontaneous indels tend to be flanked by low GC content is consistent with data on divergence between species [40]. Accounting for systematic variation in mutation rates within genomes will be necessary to develop accurate models of neutral molecular evolution, in order to correctly identify the effects of selection and other patterns of genome evolution such as codon bias [4,5]. The MA strategy combined with genome sequencing is a promising and relatively unbiased approach to identify patterns of variation in the mutational spectrum, both within and among genomes.

## Methods

### DNA Extraction and Sequencing

Following MA, we crossed pairs of independent MA lines from the same (3rd chromosome) treatment, creating genotypes that were heterozygous for new mutations, and froze these flies at –80°C. Using the Qiagen DNeasy Blood and Tissue Kit insect tissue protocol with minor modifications, we extracted genomic DNA from 56 heterozygous genotypes selected at random from seven genetic quality treatments, using at least 28 males, and on average 47 males per heterozygous genotype. We sequenced either heterozygous genotypes or pooled samples of two heterozygous genotypes so that the expected frequency of new mutations was 0.5 or 0.25, respectively. See S1 Data for coverage information. We also sequenced the stock population of flies (carrying the “*vg*” chromosome) used to maintain the MA lines, to identify gene conversion events; in this case, DNA was extracted from 200 flies, frozen at generation 52, and average coverage was 60X. Library preparation and multiplexed paired-end 100 bp sequencing was conducted at the University of British Columbia Biodiversity Research Centre (Vancouver, BC) or at The Centre for Applied Genomics (The Hospital for Sick Children, Toronto, ON), using Illumina HiSeq technology. Sequencing was conducted in four blocks (samples 1–16, 17–26, 27–36, 37–38), with additional sequencing runs performed for some blocks because of poor initial coverage.

### Calling Single-Nucleotide Substitutions and Small Indels

We conducted multiple alignment steps to reduce mapping error. Reads from each sample were mapped to the *D*. *melanogaster* reference genome (v. 5.56), using *BWA* (v. 0.5.9) [41] and *Stampy* (v. 1.0.21) [42]. Duplicate reads were removed using Picard tools (http://picard.sourceforge.net), and the data were remapped using IndelRealigner in *GATK* (v. 2.3.9) [43]. We combined data from all samples using *Samtools* (mpileup; v. 0.1.16) [44], considering only the focal chromosome. We used stringent mapping and calling procedures to avoid false positives [19,21] and attempted to account for the number of true mutations that were excluded by this procedure (false negatives), as described below.

For each site, we examined the total number of reads in each sample using an in-house *Perl* script. If sequences from duplicated loci are mapped to a single reference locus, this mapping error may lead to unusually high coverage in multiple samples and potentially the false appearance of mutations at intermediate frequency. We considered a sample to have high coverage if the number of reads was >2.5 times the median coverage for that sample across sites and discarded any site where >25% of samples had high coverage. At the remaining sites, we considered only those base calls with quality scores denoting accuracy > 99.9%. We discarded sites where >20% of samples had coverage <10. To infer the most likely state of the common ancestor at each site, we determined the majority base call of the reads in each sample, allowing for ties, and then determined the majority of those calls across samples. The site was discarded if >20% of samples had a majority allele that differed from this overall consensus, a problem that can arise from mapping error. We considered remaining sites to be “callable.”

At each callable site, we only considered those samples with coverage of at least nine, with at least one read on each strand. The number of callable sites in each sample is given in S1 Data. We recorded the forward and reverse coverage of each of these callable samples for later analyses. We identified putative substitutions in callable samples as cases with at least five nonconsensus base calls, with at least one nonconsensus base call on each strand. This and other elements of the calling criteria are sensitive to both coverage and the expected mutant frequency (25% or 50%) but are accounted for in our assessment of detection power used to estimate rates. When a putative mutation was called, we collected additional information about the focal sample, as well as other samples at the site, for further analysis. We discarded cases with evidence of mapping error (S1 Text), as well as cases where a base matched the homologous, non-MA chromosome “*vg*,” which we dealt with in the gene conversion analysis described below. Following previous authors [20], each remaining putative substitution was examined in *IGV* for other possible problems. We discarded additional cases of mismapping, which were primarily due to indels or SNPs in the consensus sequence or repetitive sequence in the reference. We tested nine putative substitutions and two putative multinucleotide mutations by Sanger sequencing and confirmed them all.

To call small indel events, we used the *Pindel* pipeline [45] following alignment with *BWA*. As with substitutions, we only retained cases where the putative indel was supported by at least five reads, with at least one supporting read on each strand. As with substitutions, a signature of mapping error was the presence of the same putative indel in multiple samples. We discarded cases where a putative indel appeared in more than five reads from other samples, or where the mutant frequency was unusually low (binomial probability <0.001), and we visually examined the remaining cases in *IGV*. We tested 21 putative indels by Sanger sequencing and confirmed them all.

### Power to Detect Substitutions and Indels

At sites not considered callable, e.g., due to high depth, our power to detect mutations is zero in all samples. Similarly, we also have zero power to detect mutations at a site in a sample where the coverage is too low, though the site could be callable in other samples. For a sample in which the site is callable with forward coverage of *n*_*F*_ > 0 and reverse coverage of *n*_*R*_ > 0, we estimated the probability of detecting a true mutation as

$$P_{detect}=\overset{n_{F}}{\underset{i=1}{\sum }}\overset{n_{R}}{\underset{j=1}{\sum }}X_{i,j}BB\left[i,n_{F},f_{mut},\rho_{nF}\right]BB\left[j,n_{R},f_{mut},\rho_{nR}\right]$$

where *X*_*i*,*j*_ = 1 if *i* + *j* ≥ 5 (the minimum number of reads required to call a mutation) and zero otherwise, *f*_*mut*_ is the expected frequency of new mutations in the sample (0.25 or 0.5), and *BB* is the beta-binomial density function with overdispersion *ρ*. *P*_*detect*_ is reduced below its maximum value of 1 as total coverage (*n*_*F*_ + *n*_*R*_) decreases, or as coverage becomes biased towards the forward or reverse strand. We incorporated overdispersion because of evidence that the number of mutant calls in a heterozygous sample can be overdispersed relative to a binomial distribution [20]. We estimated overdispersion (*ρ*) for each sequencing block by maximum likelihood (S2 Text). Estimates of *ρ* were small (1.01–1.17) and not significantly different from 1 (no overdispersion) in most blocks. Nevertheless, we used these ML values when calculating *P*_*detect*_ for subsequent analyses.

For each sample, we calculated Ω as the sum of *P*_*detect*_ across all sites and multiplied by the number of MA lines within the sample (2 or 4). Ω represents the effective number of sites called in a sample across all MA lines present in that sample (2 or 4), weighted by the relative opportunity for mutation. For example, if the data for a sample consisted of *x* sites sequenced at very high coverage (so *P*_*detect*_ ≈ 1 for these sites), then Ω ≈ 2*x* (or 4*x*) if the sample was comprised of 2 (or 4) MA lines. Detection power (*P*_*detect*_) was about half as large for samples consisting of 4 MA lines (where mutant frequency was 25%) compared to samples consisting of 2 MA lines (where mutant frequency was 50%). Consequently, Ω did not differ between samples with 2 versus 4 MA lines, reflecting the trade-off between sequencing more MA lines in a single sample and sequencing each MA line within a sample with greater coverage. As expected, the number of substitutions detected was highly correlated with Ω across samples (*r* = 0.87; *N* = 38; *p* < 1 × 10^−11^).

Although indel mutations will sometimes involve more than one site, it is likely that Ω is nevertheless a good approximation for our power to detect these events in our data, because the indels we detected were generally small (mode = 1 bp, mean = 1.9 bp, max = 4 bp), and because we applied the same coverage criteria for calling indels and substitutions, which are more stringent than the criteria used by *Pindel*. The number of indels detected was positively correlated with Ω across samples (*r*_*Spearman*_ = 0.57; *N* = 38; *p* < 0.001). We therefore used Ω to assess our power to detect indels and estimate the rate of indels. Nonetheless, we caution that Ω should be regarded as a cruder approximation of power for detection of indels than for substitutions. It will be sufficient for our main goal of comparing indel rates between loaded and unloaded backgrounds, as the method is applied to both treatments and sequence coverage levels are similar between treatments.

### Annotations of Substitutions and Indels

We downloaded gene locations and sequences from FlyBase (https://flybase.org). We simulated >27,000 random point mutations and determined our power to detect mutations at each site and whether simulated mutations occurred in genes. We also determined whether each observed substitution and indel occurring in coding sequence was synonymous or nonsynonymous. A mutation was considered nonsynonymous if it altered the amino acid sequence or length of at least one known protein isoform. Finally, we also tested whether the frequency of nonsynonymous versus synonymous substitutions differed from the neutral expectation by simulating random substitutions in the relevant genes (>1.2 × 10^6^ simulations total), assuming a transition bias of 2:1, and ignoring variation in our power to detect mutations.

### Detecting Gene Conversion Events and Assessing Power

During MA, the focal second chromosome was maintained in heterozygous males. The homologous chromosome, “*vg*,” was derived from a stock bearing recessive markers introgressed onto a wild-type background. This stock differed from the MA consensus sequence at 1 in every 272 callable sites (after weighting by alternate allele frequency), but the stock itself was genetically variable. We can detect gene conversion events if they occur in a region where the focal chromosome and the *vg* chromosome differ, because the MA chromosome will carry SNPs that are commonly found on the *vg* chromosome. Our approach to calling gene conversion events and assessing power is described in detail in S3 Text. Briefly, we first identified sites where *vg* differed from the MA consensus, which we refer to as *vg*-SNPs. We then identified sites in each MA sample with reads that matched a *vg*-SNP. Using this list of matching sites, we called a gene conversion event in a given sample when there were at least three sites, each with at least three high-quality base calls matching a *vg*-SNP, within a 5-kb window. On average, there were 10.7 matching sites per event we detected, with an average of 6.8 matching calls per site. In a separate analysis, we also applied less stringent criteria by including cases where only one site had at least five high-quality base calls matching *vg*-SNPs; our results are qualitatively unchanged by the inclusion of such cases. All putative gene conversion events were examined in *IGV*. We tested three putative gene conversion events by Sanger sequencing and confirmed them all.

To assess power, we simulated random events of various tract lengths and determined the probability that each event would be detected given our calling criteria, the expected frequency of a new mutation in the MA sample, and the list of *vg*-SNPs. Previous studies indicate that gene conversion tracts are exponentially distributed in length, with a mean of approximately 1,463 bp [24]. Although we cannot determine the exact lengths of the gene conversion events in our dataset we examined approximate lengths based on the distance between the leftmost and rightmost sites within a tract with two or more base calls matching a *vg*-SNP, which will underestimate the true length. This distribution did not differ significantly from an exponential distribution with mean 1,463 bp (see Results). We calculated power-corrected gene conversion rates assuming as exponential distribution of tract lengths with mean *λ* = 1,463 bp (see S3 Text), but our main conclusions are unchanged when values of *λ* ~30% shorter or longer (e.g., *λ* = 1,000, 1,900) are assumed instead.

### Testing for Crossing Over

In the first block of genome sequencing, we found evidence that crossing over occurred between the focal MA chromosome and the homologous *vg* chromosome in samples 1, 2, and 16. Specifically, large numbers of substitutions were identified on the left arm of the chromosome, which were of the expected mutant frequency (0.25 for these samples), and matched polymorphisms on *vg*, indicating that crossing over occurred in one of the MA lines comprising each of these samples (S2 Fig). We excluded these regions from all analyses (all of 2L for samples 1 and 2, and the distal 3 Mb in sample 16). Prior to sequencing blocks 2–4, we tested additional MA lines for crossing over with *vg* by Sanger sequencing two loci on the distal end of chromosome 2L that include nine SNPs found in the *vg* population. Based on the location of the markers used in the MA experiment, recombination effects on 2R would have been excluded during MA, whereas those on 2L would have been undetectable. In addition to the three lines in the dataset presented here, we found evidence for crossing over in two additional lines (out of 184 tests in total). We excluded these lines from genome sequencing in blocks 2–4; the additional lines chosen for sequencing were chosen more or less at random, given the availability of material. There was no genomic evidence of crossing over in the 2nd chromosomes we fully sequenced that tested negative for crossing over based on Sanger sequencing (S2 Fig).

### Calling TE Insertions and Excisions

We identified TEs in our data using *PoPoolationTE* [26], which aligns reads to known TE sequences, along with a genome reference where these sequences are masked. Read pairs that map partially to a genomic sequence and partially to a TE sequence provide an indication of the location and frequency of TEs in the genome. We downloaded TE sequences from FlyBase (http://flybase.org) and NCBI (http://www.ncbi.nlm.nih.gov). *PoPoolationTE* is known to be sensitive to the distribution of insert sizes and coverage, and our data are not ideally suited for detection of TEs. We use it here only to get a crude sense of TE activity and to test if there are obvious differences in TE activity between treatments. We identified putative TEs in each sample independently and then clustered TEs by family based on their putative locations. A novel insertion will appear at intermediate frequency in one sample only. An excision of a consensus TE sequence will appear at intermediate frequency in one sample and be fixed in the remaining samples. In addition to these criteria, we only considered putative TE insertions and excisions that were supported by at least five reads, with at least one read on each strand, and with presence/absence information from at least nine reads in total. We did not identify any TE excisions. Some of these criteria for calling TEs are similar to our criteria for calling substitutions and indels, and the number of TEs detected was significantly correlated with Ω across samples (*r* = 0.76; *N* = 38; *p* < 1 × 10^−7^), indicating that Ω may account for much of the variation among samples in our power to detect TEs. The rates of TE insertion we report are calculated by assuming that our highest value of Ω corresponds to a TE detection probability of 1 and thus represent lower bound estimates of the TE insertion rate.

### Statistical Analyses

We tested the effect of genetic quality (loaded or unloaded) on the rates of single-nucleotide substitution, indels, gene conversion events, and TE insertions by fitting GLMs with *N* = 38 samples in *R* [46]. Because our initial goal was to compare loaded and unloaded backgrounds, we do not distinguish among different types of loaded backgrounds in these analyses. We also ran these models including “background genotype” as a random effect, but the random effect term was nonsignificant. In addition to a main effect of background, we included power as a covariate to account for differences in power and the number of MA lines within each sample. Multinucleotide mutations were each treated as single events. For indels and TEs, we found that the number of MA lines within a sample had a significant effect on the observed number of events, beyond the effect of power per se, and so we included it as an additional covariate in these cases. Each model used a Poisson link function, and we tested for possible overdispersion using the *R* package *AER*. We detected marginally significant overdispersion in the case of gene conversion (*p* = 0.0497). Because opinions differ on how to test fixed effects when there is overdispersion, we fit both a quasi-Poisson model (Wald *t* test for an effect of treatment), and a generalized linear mixed model (GLMM) with an observation-level random effect (likelihood ratio test [LRT] for an effect of treatment), which gave the same conclusions. For other mutation types, we tested for an effect of treatment using a Wald *Z* test. In addition, we tested for an effect of sequencing block by incorporating a random effect of block in GLMMs, but the random effect variance for block was found to be negligible in all cases, and was not included in our final analyses. Our mutation rate estimates are based on predicted values from these models (quasi-Poisson in the case of gene conversion) for a sample with average power, accounting for power and the number of MA generations. To test for an effect of body mass on indel and gene conversion rates, we fit Poisson GLMs as above, with mass instead of treatment as a main effect. We initially included a random effect of genetic background (seven levels) to account for overdispersion, but found that the variance attributed to this factor was negligible. Further, we found that a model using only “background mass” was much better than a model using only “background genotype” based on AIC scores, suggesting that variation in mutational properties among backgrounds is more simply accounted for by their effect on mass than by their specific genotypes. For all models, detection power was a significant positive predictor of observed mutation number.

### Analysis of Genomic Context Effects on Mutation

To examine the effect of local context on substitution rates, we determined our context-specific power to detect substitutions at the central position in each of the 32 possible 3-bp contexts in each sample. We also considered the effect of GC content in a wider region on the rate of substitutions and indels by first finding the GC content of sites with substitutions, and of the 6 bp centered on the midpoint of each indel, and then determining the GC content in the 1 kb surrounding each of these focal sites or 6-bp regions (data to the left and right of each site were averaged). In addition to observed mutations (excluding multinucleotide substitutions), we conducted this procedure for >86,000 randomly chosen sites, across samples, where we simulated twice as many events for those samples representing 4 versus 2 MA lines, and retained each simulated event according to the probability that it would be detected. We divided these focal sites based on the GC content of the focal site (two categories for substitutions [GC versus AT], three categories for indels [%GC < 1/3, 1/3 ≤ %GC ≤ 1/2, %GC > 1/2]). We calculated average GC content in a 51-bp overlapping sliding window for mutant sites relative to nonmutant sites. We then plotted the weighted average across categories of focal site, with weights based on the GC content of random sites on chromosome 2. We also searched for the combination of window sizes for which GC content best predicted the presence of mutations using logistic models that also included the GC content of the focal site and a random effect of sample (preliminary tests indicated that interaction effects were absent). We determined the AIC score for models with 1, 2, or 3 nested windows, with the condition that each window was at least 1.25 times the size of the next smallest window, by first testing >1,000 random combinations of window sizes and then testing a grid of window combinations surrounding the combination with the lowest AIC score from the original random set. The best model involved three windows for substitutions and two windows for indels. To test for possible effects of chromosomal location on mutation rates, we determined our power to detect events in each of 44 ~1-Mb regions across the chromosome, based on all callable sites for substitutions and indels and 10^4^ simulated gene conversion events with tract lengths of 5 kb, and used this to calculate power-corrected mutation and gene conversion rates for each region. We then examined the rank correlation between these region-specific rates and that region’s distance from the centromere.

We also examined the GC content of gene conversion tracts in the following manner. We first determined the approximate midpoint of each observed tract as halfway between the leftmost and rightmost sites matching *vg*-SNPs, considering only sites with at least two matching calls, and examined the GC content of the consensus sequence in the 1,463 bp surrounding each midpoint, where 1,463 bp is the expected mean tract length [24]. We then simulated >28,000 gene conversion events in random locations on chromosome 2, with exponentially distributed tract lengths with mean 1,463 bp. We simulated twice as many events for those samples representing 4 versus 2 MA lines, and retained each simulated event according to the probability that it would be detected. We then compared the GC content of the 1,463 bp surrounding the midpoints of simulated gene conversion tracts to that of the observed tracts.

To assess the possibility of GC-biased gene conversion, we used *vg*-SNP sites to determine the spectrum of changes expected in gene conversion tracts in the absence of bias, accounting for the observed frequency of each *vg*-SNP, to compare with the observed spectrum (S1 Data). Our power to detect bias is limited, because the locations of heteroduplex regions of gene conversion tracts are uncertain, and we cannot detect cases where mismatches are resolved in favour of the consensus base.

## Supporting Information

S1 Data Data underlying plots and additional information. (XLSX)

S1 Fig Comparison of single-nucleotide substitution spectrum between the present study and the two genetic backgrounds studied by Schrider et al. [19]. The distribution of substitutions we observed was significantly different from both line 39 (χ^2^ = 12.86, *df* = 5, *p* < 0.03) and line 33 (χ^2^ = 40.77, *df* = 5, *p* < 1 x 10^−6^). See S1 Data for plot data.
(PDF)

S2 Fig Evidence for crossing over in some samples. Blue shading for each sample represents the fraction of sites within nonoverlapping 100-kb windows with at least three reads matching a *vg*-SNP (in most samples these values are negligible). The distance between horizontal lines represents 1% of sites in a given window. Extensive matches in samples 1, 2, and 16 indicate crossing over occurred on 2L in these samples. Of all matches in samples 1 or 2, 53% occur in both samples. Of the matches in sample 16, 37% also occur in sample 1 or 2. Prior to genome sequencing samples 17–38, we excluded lines where crossing over was detected based on Sanger sequencing (supplementary text), which is consistent with the lack of crossing over detected in these genome sequences.
(PDF)

S1 Text Methods for identifying cases of mapping error in calling single-nucleotide substitutions. (PDF)

S2 Text Methods for estimating overdispersion to calculate *P*_*detect*_. (PDF)

S3 Text Methods for detecting gene conversion events and assessing power. (PDF)

S4 Text Methods for reanalysis of MA fitness data. (PDF)

S5 Text Methods for maximum likelihood analysis of DNA repair. (PDF)

## Abbreviations

AIC: Akaike information criterion
CI: confidence interval
DSB: double-strand break
GC: guanine-cytosine
GLM: generalized linear model
GLMM: generalized linear mixed model
HR-h: homologous recombinational repair using homolog
indel: insertion or deletion
LRT: likelihood ratio test
MA: mutation accumulation
NHEJ: nonhomologous end-joining
SSA: single-strand annealing
TE: transposable element
