# Supplementary figures and images for: Low Genetic Quality Alters Key Dimensions of the Mutational Spectrum
Source: PLoS Biol. 2016 Mar 25;14(3):e1002419. doi: 10.1371/journal.pbio.1002419 (PMC4807879; doi:10.1371/journal.pbio.1002419)

Proportion of substitutions

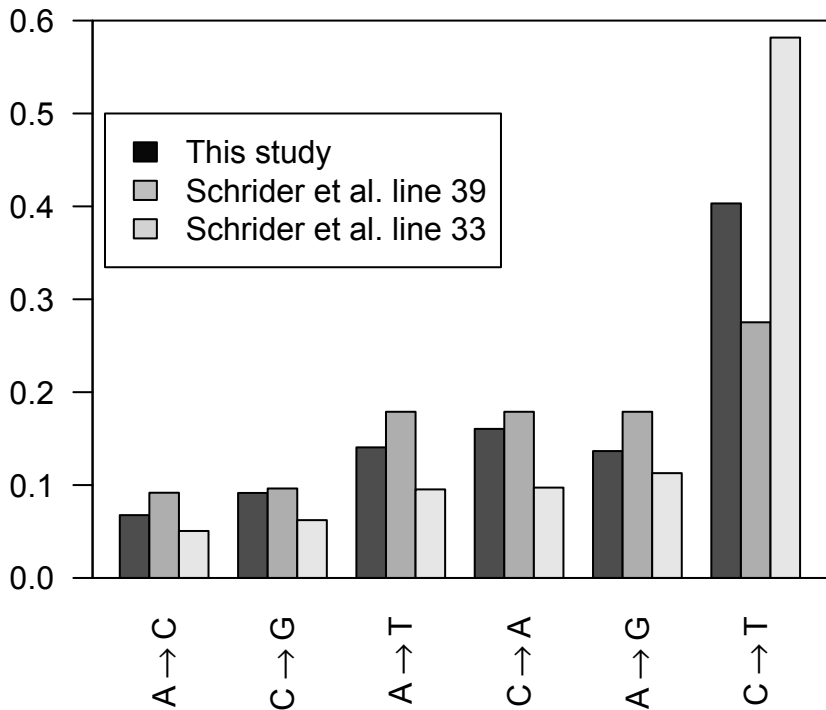

Supplement: S1 Fig — The distribution of substitutions we observed was significantly different from both line 39 (χ2 = 12.86, df = 5, p < 0.03) and line 33 (χ2 = 40.77, df = 5, p < 1 x 10^−6^). See S1 Data for plot data. (PDF) [file pbio.1002419.s002.pdf]

Sample

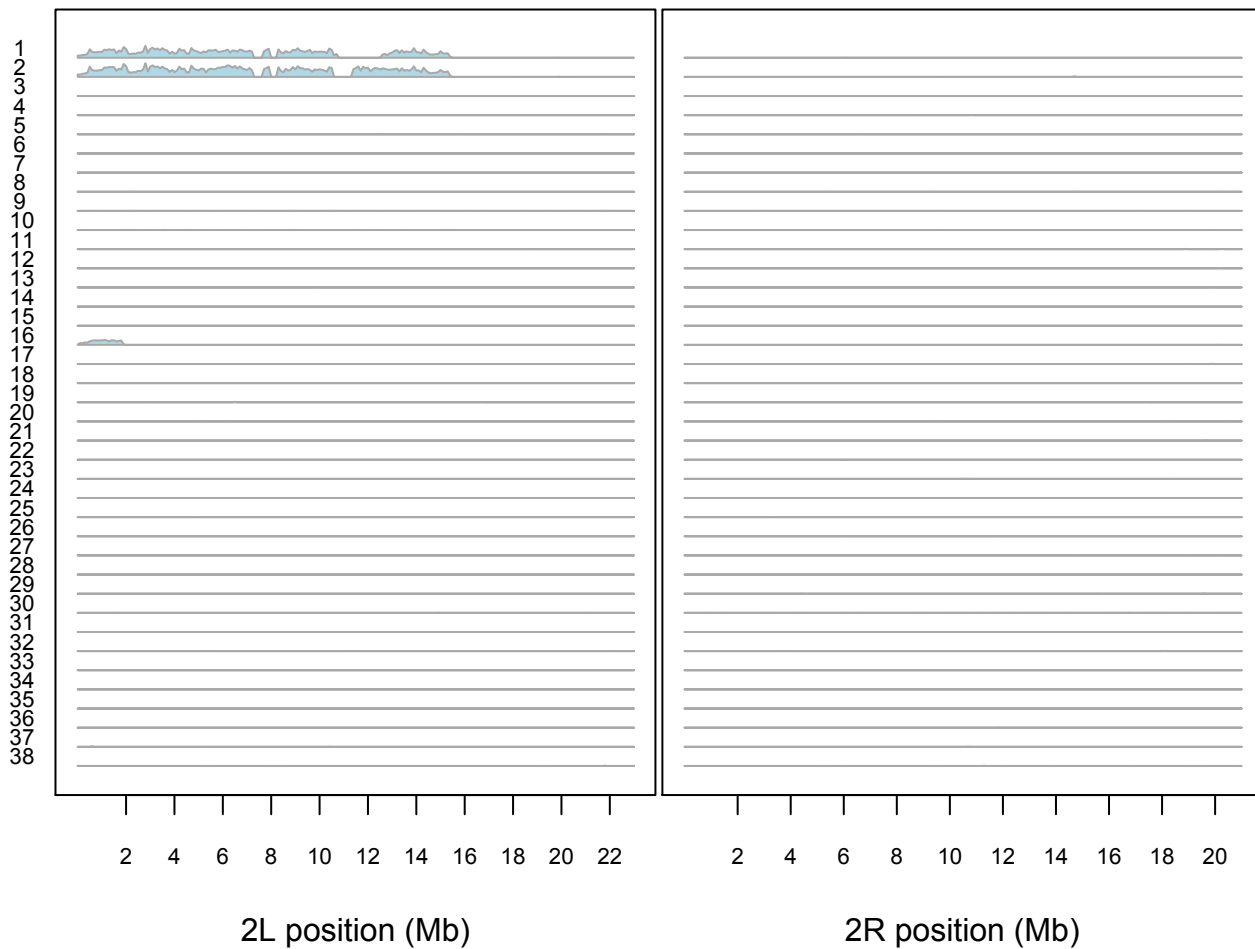

Supplement: S2 Fig — Blue shading for each sample represents the fraction of sites within nonoverlapping 100-kb windows with at least three reads matching a vg-SNP (in most samples these values are negligible). The distance between horizontal lines represents 1% of sites in a given window. Extensive matches in samples 1, 2, and 16 indicate crossing over occurred on 2L in these samples. Of all matches in samples 1 or 2, 53% occur in both samples. Of the matches in sample 16, 37% also occur in sample 1 or 2. Prior to genome sequencing samples 17–38, we excluded lines where crossing over was detected based on Sanger sequencing (supplementary text), which is consistent with the lack of crossing over detected in these genome sequences. (PDF) [file pbio.1002419.s003.pdf]
