# Supplementary material for: Low Genetic Quality Alters Key Dimensions of the Mutational Spectrum
Source: PLoS Biol. 2016 Mar 25;14(3):e1002419. doi: 10.1371/journal.pbio.1002419 (PMC4807879; doi:10.1371/journal.pbio.1002419)
Supplement: S1 Text — (PDF) [file pbio.1002419.s004.pdf]

S1 Text: Identifying cases of mapping error in calling single-nucleotide substitutions.

We examined the data on putative substitutions, and found evidence that some were the result of mapping error, which can be a major source of false positives in mutation detection [1]. The primary evidence for mapping error was that a putative mutant base was present in multiple samples. It is extremely unlikely that the same mutation would occur independently in multiple MA lines [2]. The large number of samples in our dataset, which all originated from the same ancestral chromosome, provided us with high power to identify these cases. The presence of a putative mutation in multiple samples was associated with lower quality scores, fewer callable samples, a higher degree of strand bias, and unusually high or low mutant frequency in the focal sample. Based on this analysis, we considered putative mutant bases that were present in > 1% of reads in the non-focal samples to be suspicious. For a subset of suspicious cases, we visually examined the alignment using the Interactive Genome Viewer *IGV*, v. 2.3.26 [3] and found clear indications of mapping error due to indels in the consensus relative to the reference, or repetitive sequence in the reference. We therefore discarded all suspicious cases. We also discarded cases where the frequency of the mutant allele was unexpectedly low, given total coverage in that sample (binomial probability < 0.001).

References

1. Keightley PD, Ness RW, Halligan DL, Haddrill PR. Estimation of the spontaneous mutation rate per nucleotide site in a *Drosophila melanogaster* full-sib family. Genetics. 2014;196: 313–320. doi:10.1534/genetics.113.158758

2. Zhu YO, Siegal ML, Hall DW, Petrov DA. Precise estimates of mutation rate and spectrum in yeast. Proc Natl Acad Sci USA. 2014;111: E2310–E2318. doi:10.1073/pnas.1323011111

3. Robinson JT, Thorvaldsdóttir H, Winckler W, Guttman M, Lander ES, Getz G, et al. Integrative genomics viewer. Nature Biotech. 2011;29: 24–26. doi:10.1038/nbt.1754
