# Supplementary material for: Low Genetic Quality Alters Key Dimensions of the Mutational Spectrum
Source: PLoS Biol. 2016 Mar 25;14(3):e1002419. doi: 10.1371/journal.pbio.1002419 (PMC4807879; doi:10.1371/journal.pbio.1002419)
Supplement: S2 Text — (PDF) [file pbio.1002419.s005.pdf]

S2 Text: Estimating overdispersion to calculate P_{detect}.

Based on an exploratory analysis of raw data on heterozygous mutations from another study (generously provided by P.D. Keightley), we chose to model overdispersion (ρ) in our data set as a function of total coverage (n), approaching zero as coverage increases: $\rho(n|\sigma) = (\sigma - 1)/(n - 1)$, such that the variance with overdispersion is σ times greater than the variance in the absence of overdispersion. (When $n = 1$ overdispersion is not applicable, and we set $\rho = 0$.) We used our data on point mutations to find the maximum likelihood value for σ for each block. For a given case with mutant forward and reverse coverage of m_F and m_R, respectively, total forward and reverse coverage of n_F and n_R, respectively, and expected mutant frequency of p^*,

$$L(\sigma) = \frac{BB\left(m_F \middle| n_F, p^*, \rho(n_F|\sigma)\right) \cdot BB\left(m_R \middle| n_R, p^*, \rho(n_R|\sigma)\right)}{\sum_{i=1}^{n_F} \sum_{j=1}^{n_R} X_{i,j} G_{i,j,n_F,n_R} BB\left(i \middle| n_F, p^*, \rho(n_F|\sigma)\right) \cdot BB\left(j \middle| n_R, p^*, \rho(n_R|\sigma)\right)}$$

where $X_{i,j} = 1$ if $i + j \geq 5$ (the minimum number of reads to call a mutation) and zero otherwise, and $G_{i,j,n_F,n_R} = 1$ if $PB(i + j \mid n_F + n_R) > 0.001$ and zero otherwise; BB is the beta-binomial density function, and PB is the binomial probability function.
