# Supplementary material for: Low Genetic Quality Alters Key Dimensions of the Mutational Spectrum
Source: PLoS Biol. 2016 Mar 25;14(3):e1002419. doi: 10.1371/journal.pbio.1002419 (PMC4807879; doi:10.1371/journal.pbio.1002419)
Supplement: S3 Text — (PDF) [file pbio.1002419.s006.pdf]

S3 Text: Detecting gene conversion events and assessing power.

We first created a list of all sites on chromosome 2 with at least 4 high-quality base calls in *vg* that differed from the MA consensus, or 3 calls if the alternate base was the reference base (*vg*-SNPs). We then identified sites in each MA sample with one or more high-quality base calls matching a *vg*-SNP, excluding cases where the non-consensus base was present in >3 MA samples as probable mapping errors. Using this list of matching sites, we called a gene conversion event in a given MA sample when there were at least 3 sites, each with at least 3 high-quality base calls matching a *vg*-SNP, within a 5 kb window.

To assess our power to detect gene conversion events using these criteria, we simulated events of tract length L at random locations (see below for values of L used). For each simulated gene conversion tract j in each MA sample, $p_{vg,i,j}$ is the frequency of the non-consensus base in *vg* at location i, $n_{MA,i,j}$ is the actual depth of coverage in the MA sample at location i, and k is the number of *vg*-SNPs that would occur in tract j. We do not know the true frequency of *vg*-SNPs within the *vg* stock but we used the estimate based on our sequence data from *vg*. For each site i within the simulated tract j, we then calculated the number of *vg*-SNP calls expected to appear in the MA sample as $z_{i,j} = p_{vg,i,j}\, n_{MA,i,j}\, p^*$, where p^* is the expected frequency of a new mutation in the MA sample (0.25 or 0.50). To simplify our estimation of power, we made several further approximations. In cases where at least 3 sites in the simulated tract had $z_{i,j} > 3$ and $\sum_i^k z_{i,j} > 15$, we approximated our power to detect such a gene conversion as $P_{detect,L,j} = 1$. When $k < 3$ or $\sum_i^k z_{i,j} < 2$, we approximated $P_{detect,L,j}$ as 0. In all other cases, we conducted 10^4 sub-simulations, where the number of calls matching the *vg*-SNP base at location i was $B(n = 1, p = p_{vg,i,j}) \times B(n = n_{MA,i,j}, p = p^*)$, where B is a random binomial deviate. (This procedure is not strictly correct because it assumes that the frequency of observed *vg*-SNPs in a sample is independent across sites, which is untrue because of linkage disequilibrium. Nonetheless, we use this as a first-order approximation of power.) $P_{detect,L,j}$ was then given by the proportion of sub-simulations where our calling criteria were met. Our overall probability of detecting a gene conversion event of length L in a given MA sample was then $P_{detect,L} = E[P_{detect,L,j}]$, where the expectation is over gene conversions simulated in 10^4 locations.

We determined $P_{detect,L}$ for tract lengths of 50, 100, 200, 500, 1000, 1500, 2000, 5000, 7500 and 10000 bp, with 10000 random locations simulated for each. We found that the relationship between P_{detect} and tract length could be modeled in the form $P_{detect,L} = (LK)/(L + 1/cK)$. We used maximum likelihood to find the best-fit values for c and K for each sample, and then approximated the total power to detect

gene conversions assuming an exponential distribution of tract lengths as P_{detect} = $\int_0^\infty DEXP(L|\lambda)P_{detect,L}dL$, where $DEXP$ is the exponential density function with mean λ. We present results assuming a mean tract length of λ = 1463, as described in the main text.
