# Supplementary material for: Low Genetic Quality Alters Key Dimensions of the Mutational Spectrum
Source: PLoS Biol. 2016 Mar 25;14(3):e1002419. doi: 10.1371/journal.pbio.1002419 (PMC4807879; doi:10.1371/journal.pbio.1002419)
Supplement: S4 Text — (PDF) [file pbio.1002419.s007.pdf]

S4 Text: Reanalysis of MA fitness data.

Although crossing over with *vg* occurred in <3% of MA lines, genetic exchange with a non-MA chromosome presumably affected the fitness estimates for those lines, by introducing standing variation from the *vg* population. To investigate the possible effects of exchange with *vg* on our previous results [1], we re-analyzed our fitness estimates considering that four unloaded lines and one loaded line showed evidence of crossing over. We were able to determine the specific unloaded lines where crossing over occurred, but for the loaded line we know only that it was one of four lines in the pooled sample (sample 16; we did not have homozygous samples of all lines available for testing). Of the unloaded lines, only three were included in the final analysis on homozygous fitness effects, as one was recessive lethal as of generation 46. Compared to unloaded lines in the final dataset with no evidence of crossing over (N = 22), the estimated rate of fitness decline was almost 14 times more rapid in the three lines with crossing over (t = 4.34, $P < 0.001$), suggesting that exchange with *vg* had negative rather than positive fitness effects.

If crossing over reduced fitness, and crossing over occurred at a greater rate in unloaded lines, this implies that our original findings were conservative with respect to the difference between loaded and unloaded lines. As a consequence, excluding the unloaded lines with crossing over would strengthen the conclusions of our original analysis. This reanalysis could be anti-conservative if lines with crossing over are less fit than those without, since it might include loaded lines that experienced crossing over that we did not test (we tested almost all of the unloaded lines for crossing over but only 132/170 loaded lines). However, the rate of crossing over among loaded lines we tested suggests that there would be very few such cases in our final data set of lines present at all three assay time points (0.75% of 170 lines). It therefore appears highly unlikely that crossing over was the cause of the differences in fitness decline we observed between loaded and unloaded backgrounds.

As predicted above, excluding lines with any evidence of crossing over (four known unloaded lines and four loaded lines of which only one actually experienced crossing over) strengthens our previous interpretation of the fitness data. For example, our original analysis indicated that fitness decline was ~2.9 times faster in the loaded treatments; in the reanalysis this difference increases to ~7.4 times, and remains significant (permutation test, $P < 0.001$). Similarly, we originally found that the increase in among-line variance was 3.6 times faster in the loaded treatments, and this difference is ~17.0 times in the reanalysis (simulation results, $P < 0.0001$).

References

1. Sharp NP, Agrawal AF. Evidence for elevated mutation rates in low-quality genotypes. Proc Natl Acad Sci USA. 2012;109: 6142–6146. doi:10.1073/pnas.1118918109
