# Supplementary material for: Low Genetic Quality Alters Key Dimensions of the Mutational Spectrum
Source: PLoS Biol. 2016 Mar 25;14(3):e1002419. doi: 10.1371/journal.pbio.1002419 (PMC4807879; doi:10.1371/journal.pbio.1002419)
Supplement: S5 Text — (PDF) [file pbio.1002419.s008.pdf]

S5 Text: Maximum likelihood analysis of DNA repair.

If genetic quality also affects the rate of DSBs, rather than just repair pathway usage, this might lead to a difference in the total number of indel or gene conversion events between treatments. To explore the rates of DSBs and rates of usage of different DNA repair pathways, we optimized the following likelihood function, which assumes that all indel and gene conversion events are the result of DSB repair:

$$\sum_i log\left(DPois\left(obs_{GC,i}|(r_{DSB}+x\Delta_{DSB})(p_{GC}+x\Delta_{GC})\Omega_{GC,i}t\right)\right) + log\left(DPois\left(obs_{indel,i}|(r_{DSB}+x\Delta_{DSB})(1-(p_{GC}+x\Delta_{GC}))\Omega_{indel,i}t\right)\right)$$

where the summation is over all lines i, *DPois* is the Poisson density function, $obs_{GC,i}$ is the number of gene conversion events observed in sample i, r_{DSB} is the rate of DSBs in unloaded lines, x is an indicator of treatment (0 = unloaded, 1 = loaded), Δ_{DSB} is the difference between treatments in the rate of DSBs, p_{GC} is the rate DSBs are repaired via gene conversion, Δ_{GC} is the difference between treatments in the rate of repair via homologous recombinational repair (gene conversion), Ω is our probability of detecting a mutation of a given type in a given sample, and t = 52 is the number of MA generations. We optimized the function using multiple random starting values for r_{DSB}, Δ_{DSB}, p_{GC} and Δ_{GC}, and fixed Δ_{DSB} or Δ_{GC} at zero to test for an effect of treatment on the rate of DSBs and the rate of repair by gene conversion, respectively. We find that the rate of DSBs does not differ significantly between loaded and unloaded backgrounds (allowing Δ_{GC} to vary, LRT for $\Delta_{DSB} \neq 0$: D = 2.08, P = 0.15; fixing Δ_{GC} at 0, LRT for $\Delta_{DSB} \neq 0$: D = 0.68, P = 0.41), and confirm that repair leading to gene conversion occurs at a significantly higher rate in unloaded backgrounds (allowing Δ_{DSB} to vary: LRT for $\Delta_{GC} \neq 0$: D = 11.84, P < 0.001; fixing Δ_{DSB} at 0, LRT for $\Delta_{GC} \neq 0$: D = 10.43, P < 0.01, mean gene conversion repair frequency: unloaded: 0.69, 95% CI = 0.54 – 0.81, loaded: 0.35, 95% CI = 0.22 – 0.49).
